# Supplementary material for: Augmenting light coverage for photosynthesis through YFP-enhanced charge separation at the Rhodobacter sphaeroides reaction centre
Source: Nat Commun. 2017 Jan 5;8:13972. doi: 10.1038/ncomms13972 (PMC5512671; doi:10.1038/ncomms13972)
Supplement: Supplementary Information — Supplementary Figures and Supplementary References [file ncomms13972-s1.pdf]

Supplementary information

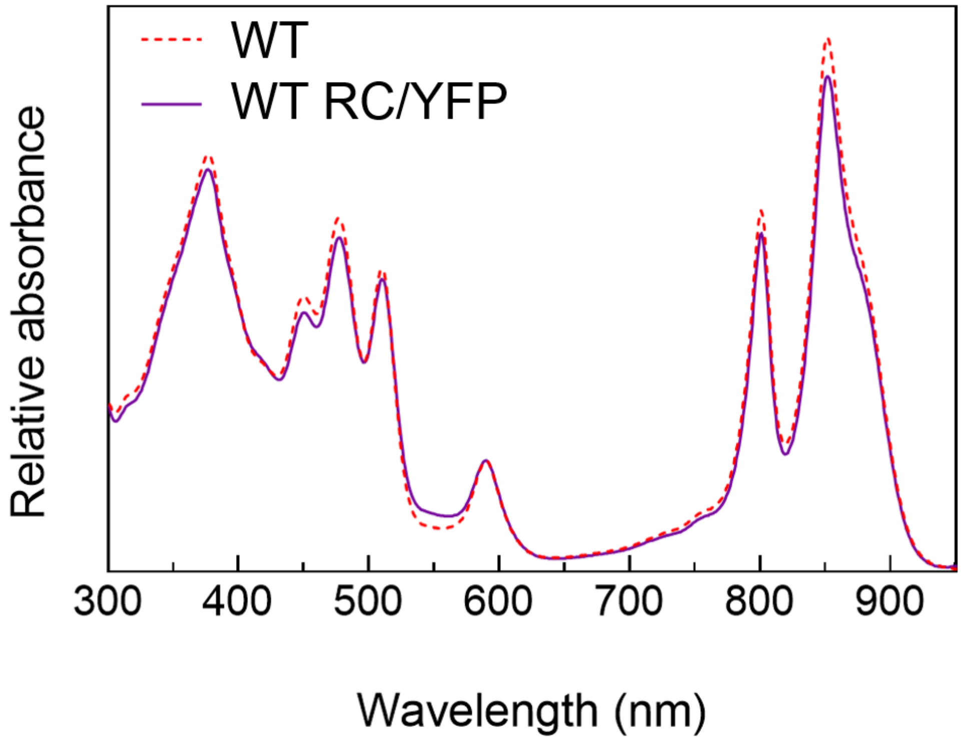

Figure S1. Room temperature absorption spectra of membranes from WT (red) and WT RC/YFP (purple) normalised to 590 nm. The RC/YFP fusion was created in a wild type *Rba. sphaeroides* background. Absorption spectra of membranes from this strain recorded at room temperature show no isolated YFP peak due to overlap with the (0-0) vibronic band of the $S_0 \rightarrow S_2$ absorption of carotenoid spheroidene at 514 nm.

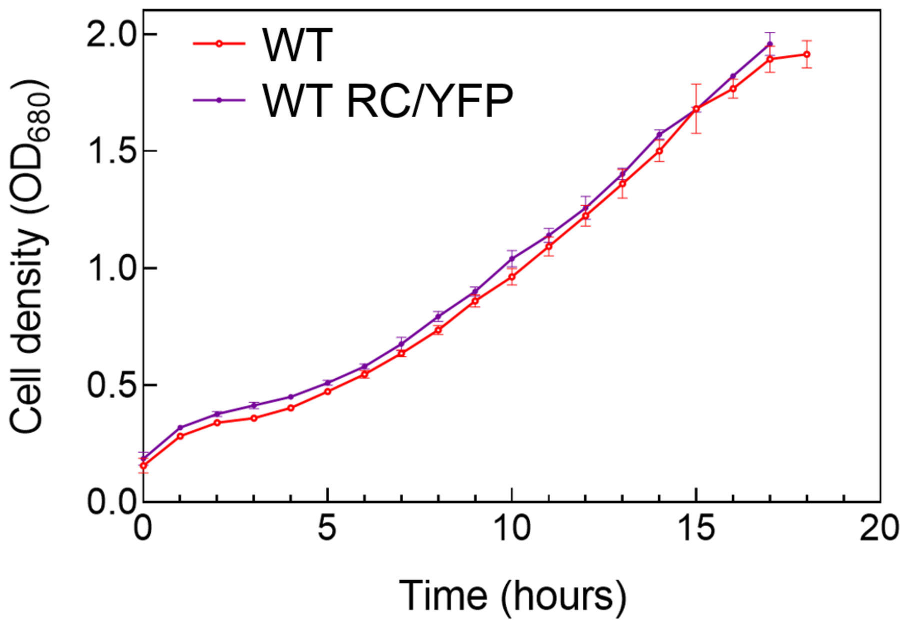

Figure S2. Photosynthetic growth curves of WT (red) and WT RC/YFP (purple). Each data point is an average from three replicates. Light was provided using Osram Halogen Eco bulbs at an intensity of 100 µmol photons s^{-1} m^{-2}.

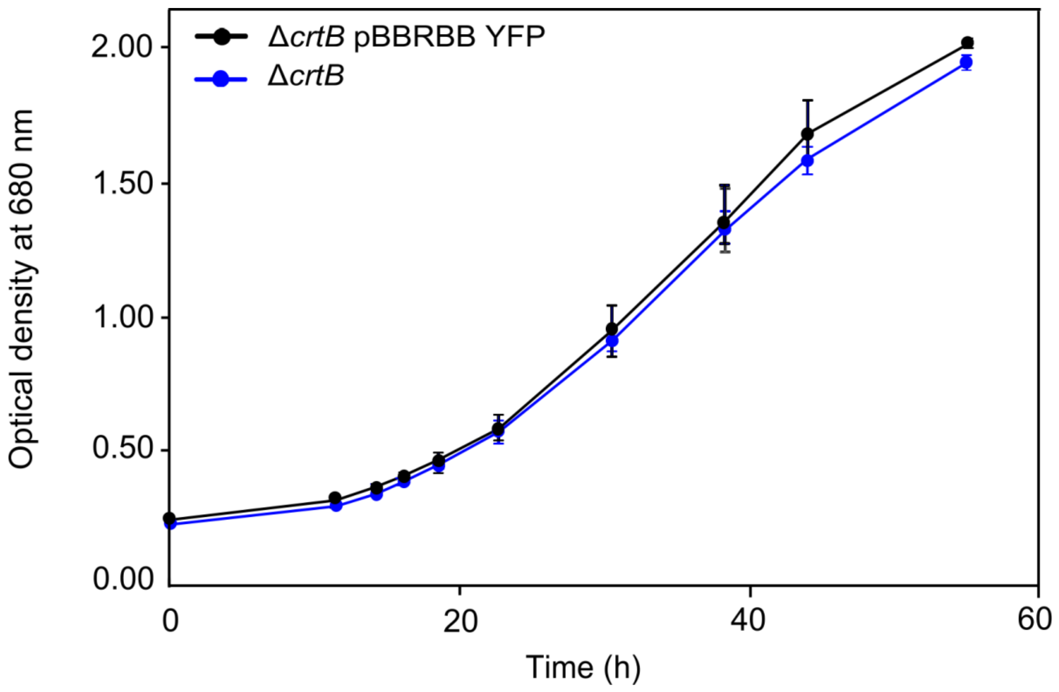

Figure S3. Photosynthetic growth curves of of Δ*crtB* (blue) and of Δ*crtB* pBBRBB YFP (black). Each data point is an average from three replicates. Light was provided using Osram Halogen Eco bulbs at an intensity of 100 µmol photons s^{-1} m^{-2}.

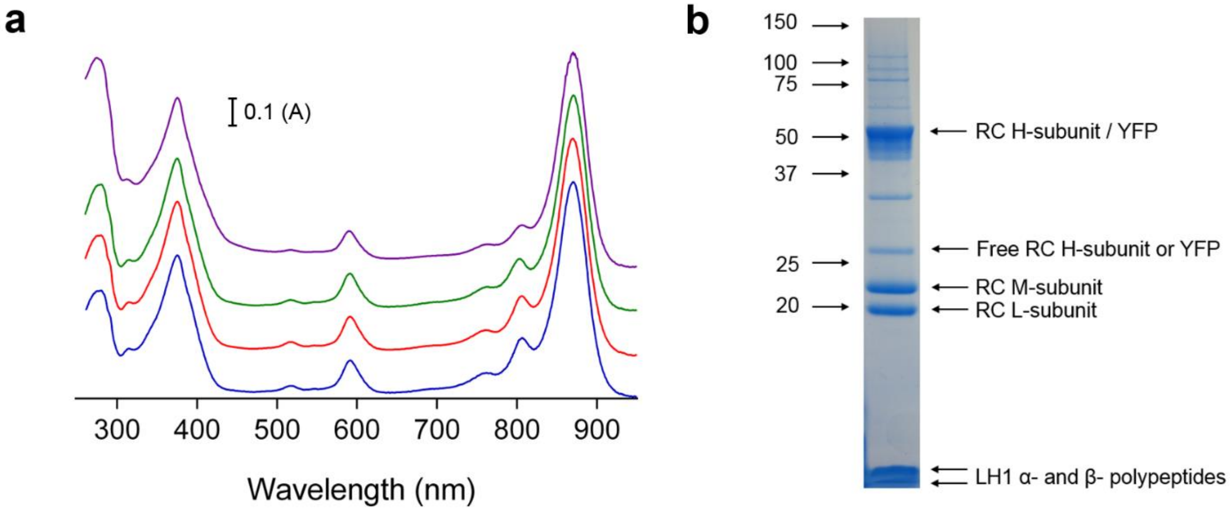

Figure S4. Purification of RC/YFP-LH1 complexes from *Rba. sphaeroides* Δ*crtB* RC/YFP-LH1

(**a**) Absorption spectra of stages in purification; A_{875}:A_{280} nm ratios are indicated in brackets alongside each sample as indicators of sample purity.

Purple	ICM	(1)
Green	Core complexes harvested from multistep sucrose gradients	(1.63)
Red	Sample after DEAE Sepharose purification	(1.73)
Blue	Sample after gel filtration	(1.92)

(**b**) SDS-PAGE analysis of purified sample after gel filtration. Molecular masses of standards are shown on the left in kDa.

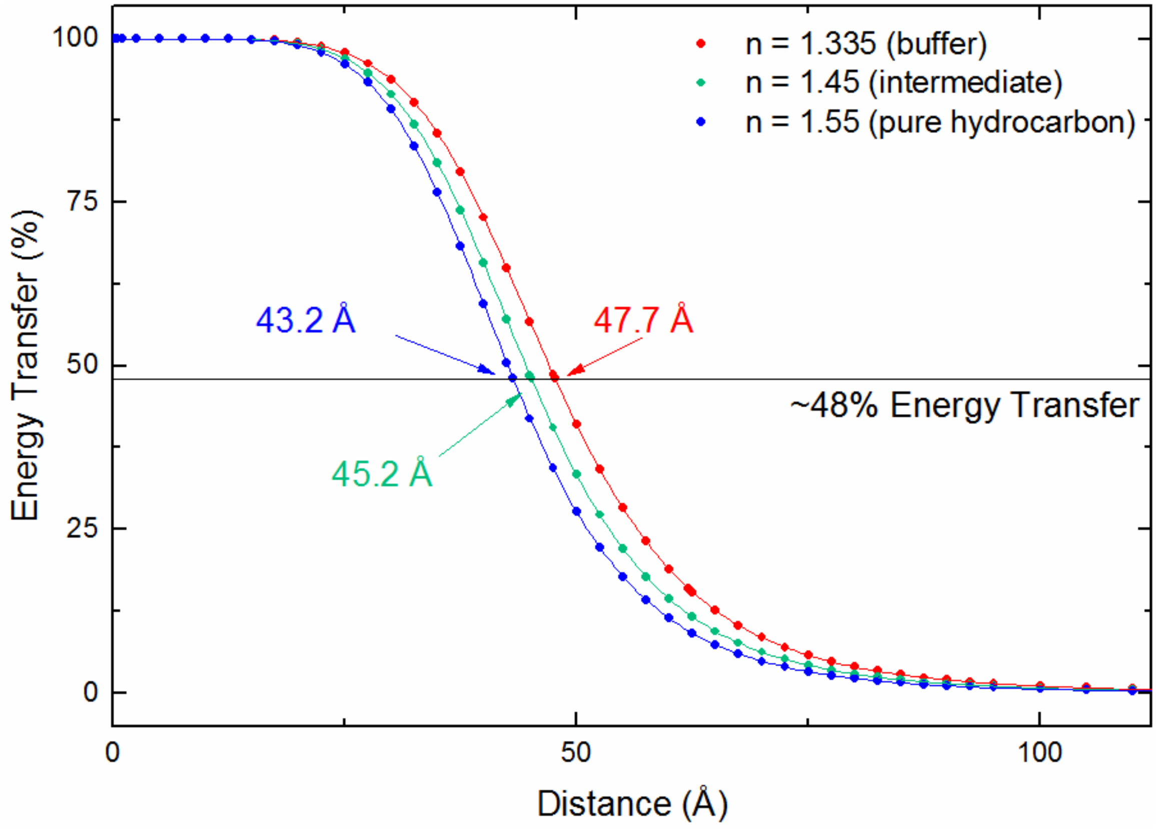

Figure S5. Estimations of the effective distance between the YFP chromophore and the RC/LH1 cofactors using Förster theory. The calculations employed PhotochemCad (Du et al., 1998) using photophysical parameters for the energy donor (YFP) and acceptor (RC). Three values of the refractive index (1.335, 1.45, and 1.55) were used to take into account the polarity of the cofactor/protein environments. The calculations, based on an average energy transfer of 48%, give a YFP to LH1 distance of 43 to 48 Å.

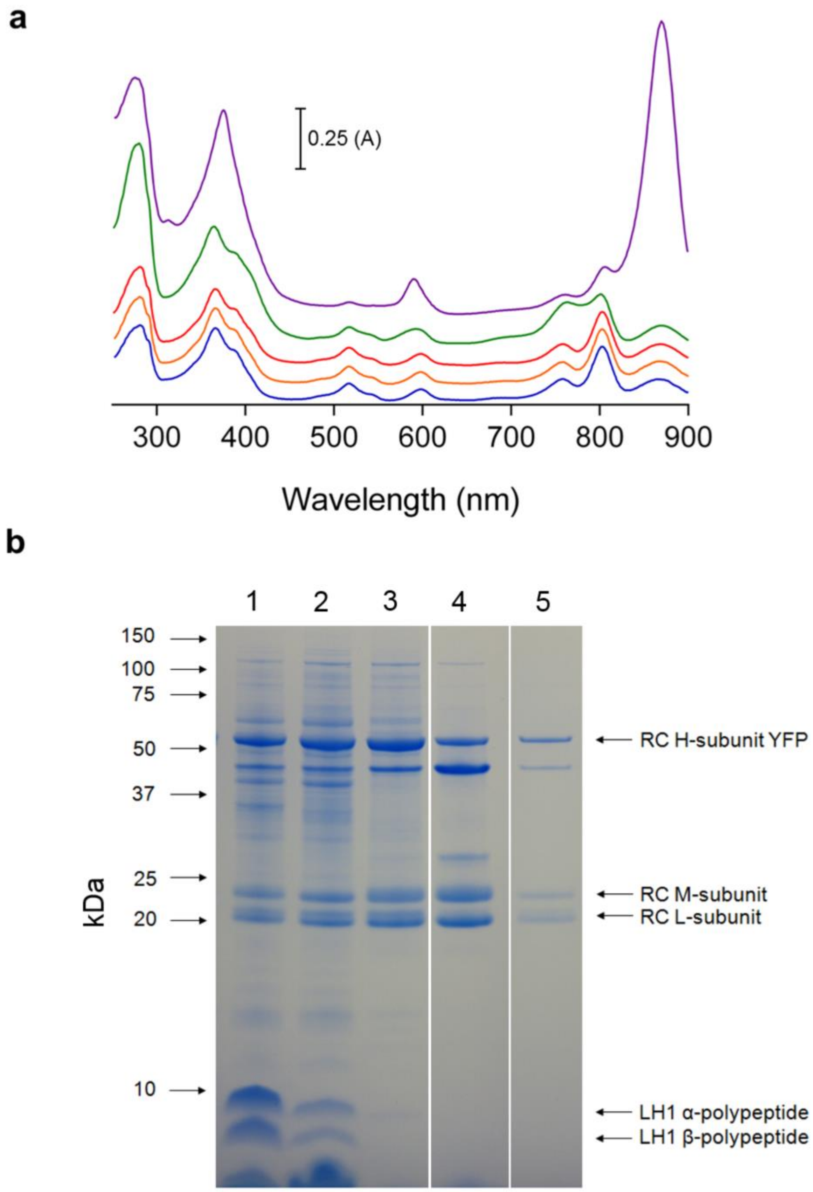

Figure S6. Purification of RC/YFP complexes from *Rba. sphaeroides* Δ*crtB* RC/YFP-LH1

(**a**) Absorption spectra of stages in purification

Purple	ICM
Green	LDAO solubilised membranes
Red	Sample after DEAE Sepharose purification
Orange	Sample after Q Sepharose purification
Blue	Purified RC/YFP after gel filtration.

(**b**) SDS-PAGE analysis of stages in purification

Lane 1	ICM
Lane 2	LDAO-solubilised complexes
Lane 3	Sample after DEAE Sepharose purification
Lane 4	Sample after Q Sepharose purification
Lane 5	Sample after gel filtration

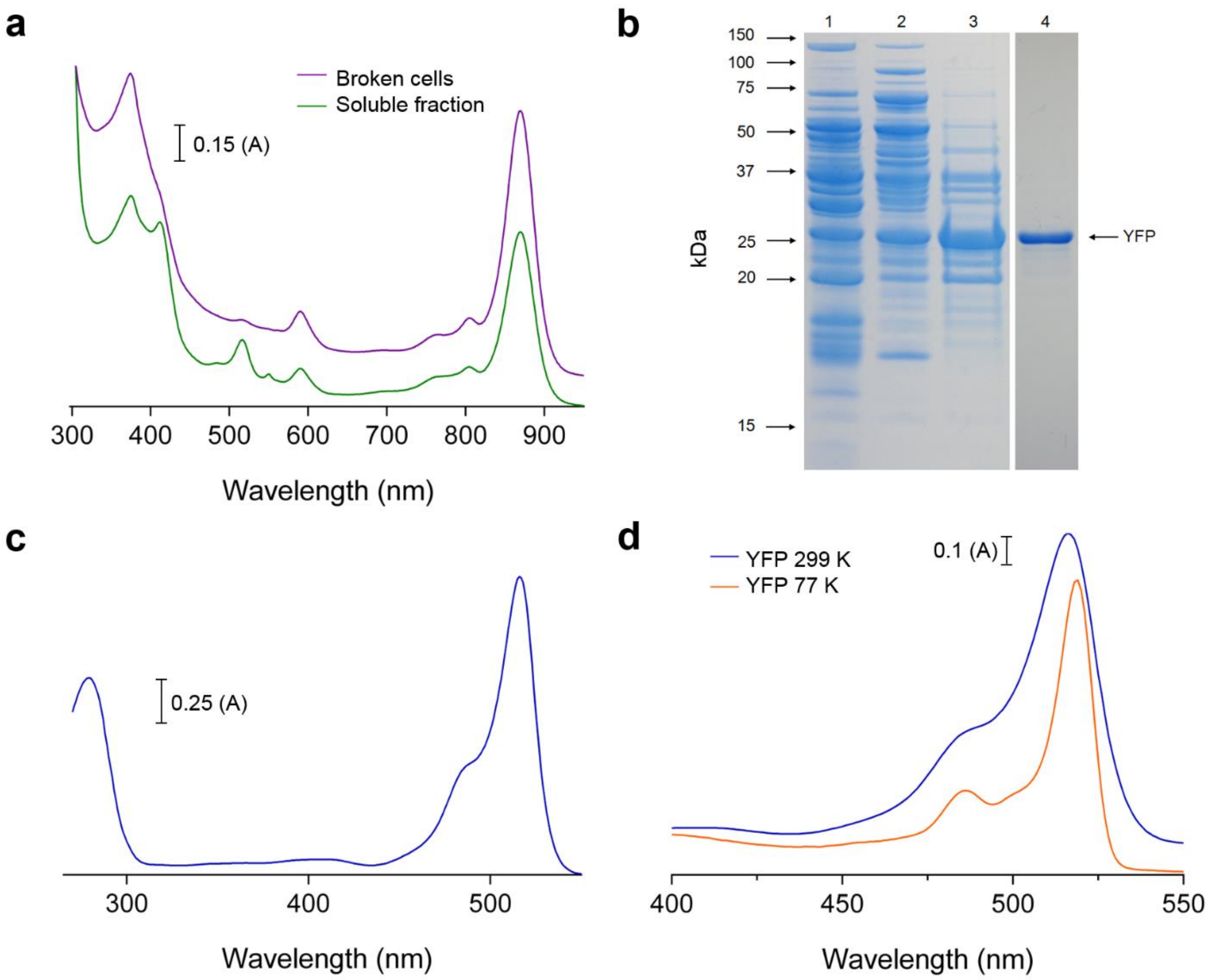

Figure S7. Purification of YFP from *Rba. sphaeroides* Δ*crtB* pBBRBB-YFP

(**a**) Absorption spectra of stages in purification

Purple Broken cells

Green Soluble fraction

(**b**) SDS-PAGE analysis of stages in purification

Lane 1 Sample after DEAE Sepharose purification

Lane 2 Sample after Q Sepharose purification

Lane 3 Sample after Gel Filtration purification

Lane 4 Final sample

(**c**) Room temperature absorption spectrum of purified YFP

(**d**) Room temperature (approximately 299 K) and 77 K absorption spectra of purified YFP

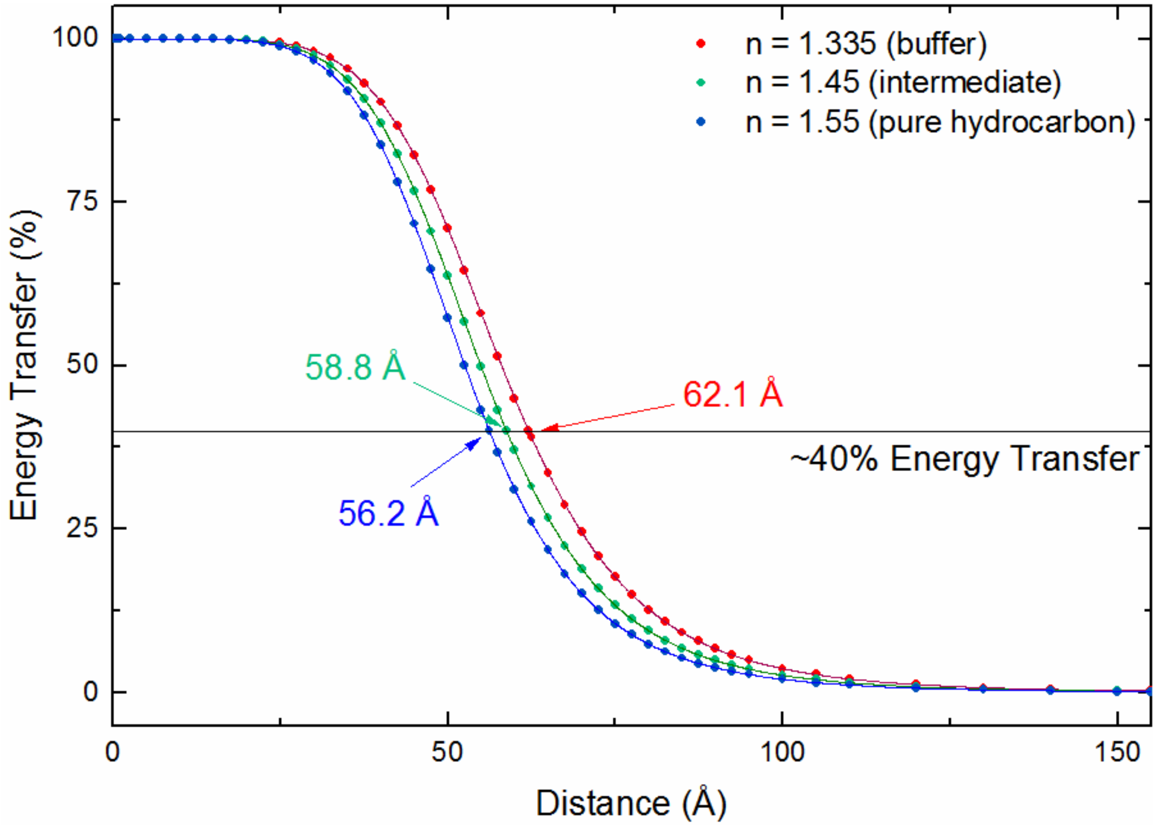

Figure S8. Estimations of the effective distance between the YFP chromophore and the RC cofactors using Förster theory. The calculations employed PhotochemCad (Du et al., 1998) using photophysical parameters for the energy donor (YFP) and acceptor (RC). Three values of the refractive index (1.335, 1.45, and 1.55) were used to take into account the polarity of the cofactor/protein environments. The calculations return a spectral-overlap integral of $J = 2.96\times10^{-13}$ cm^6, a Förster radius of R_0 = 53–58 Å, and a YFP to RC distance of 56 to 62 Å.

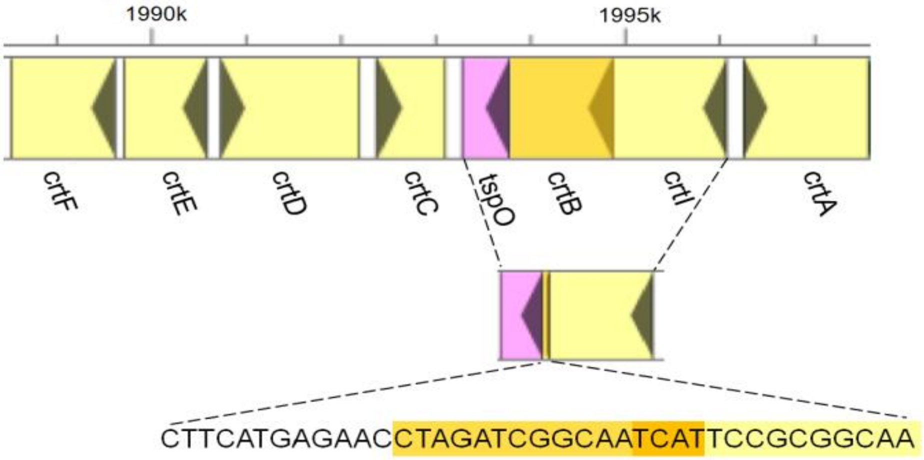

Figure S9. Deletion of *crtB* using the pK18mob*sacB* suicide vector

Schematic demonstrating the modified DNA sequence of the *crtB* deletion strain. All but the first 7 and last 9 bp of the 1068 bp *crtB* gene have been deleted.

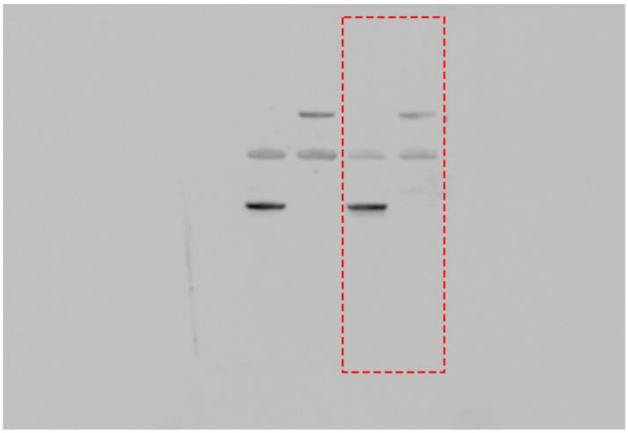

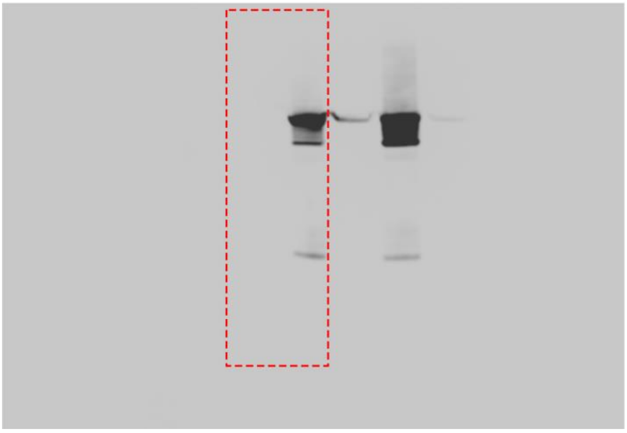

Figure S10. Uncropped immunoblots corresponding to Fig. 1a. In the upper panel antibodies specific for the RC-H subunit were used to probe Δ*crtB* and Δ*crtB*RC/YFP-LH1 membranes at two different dilutions. The red dashed box delineates the part of the image used for Fig. 1a. In the lower panel the same samples were probed with antibodies to YFP.

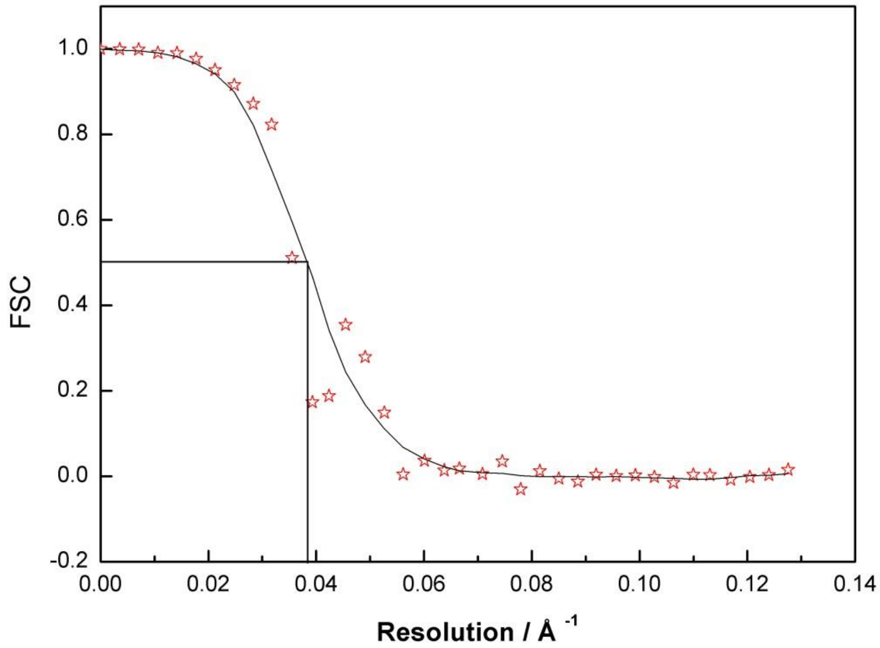

Figure S11. Fourier shell correlation calculation for the final model of the RC/YFP-LH1 complex. The stars indicate the calculated points; the solid line was fitted to these data, and yielded a resolution of 25.9 Å for the final structural model.

References

Du, H., Fuh, R.-C. A., Li, J., Corkan, L. A. & Lindsey J. S. PhotochemCAD: A computer-aided design and research tool in photochemistry and photobiology. *Photochem. Photobiol*. **68,** 141–142 (1998).
